# Latent Trajectories of Change in Dietary Restriction During Treatment in Avoidant/Restrictive Food Intake Disorder and Anorexia Nervosa

**DOI:** 10.1002/eat.24382

**Published:** 2025-01-20

**Authors:** Sophie R. Abber, Emily K. Presseller, Brianne N. Richson, Thomas E. Joiner, Christina E. Wierenga

**Affiliations:** ^1^ Department of Psychology Florida State University Tallahassee Florida USA; ^2^ Department of Psychiatry University of California San Diego Health San Diego California USA; ^3^ Department of Psychological and Brain Sciences Drexel University Philadelphia Pennsylvania USA; ^4^ Sanford Center for Biobehavioral Research Sanford Research Fargo North Dakota USA; ^5^ Department of Psychiatry and Behavioral Science University of North Dakota School of Medicine and Health Sciences Fargo North Dakota USA

**Keywords:** anorexia nervosa, avoidant/restrictive food intake disorder, classification

## Abstract

**Objective:**

Outcomes for low‐weight restrictive eating disorders, including anorexia nervosa, restricting type (AN‐R) and avoidant/restrictive food intake disorder (ARFID), are sub‐optimal. Reducing dietary restriction is a key treatment target. Understanding heterogeneity in patterns of change in dietary restriction may aid in improving outcomes. We examined latent trajectories of change in dietary restriction during treatment and follow‐up in AN‐R and ARFID.

**Methods:**

Adolescents and adults with R‐EDs (*N* = 276, 18% ARFID, 90% female, *M*_age_ = 18) receiving intensive ED treatment completed assessments at five timepoints. Latent growth mixture modeling examined trajectories of change in dietary restriction, measured using the Eating Pathology Symptoms Inventory Restricting subscale. Classes were compared on clinical features at admission to determine characteristics prospectively associated with trajectory.

**Results:**

A 3‐class solution emerged: Class 1 comprising individuals with “good response” (*n* = 138; 33% of those with ARFID in the sample); Class 2 with “good response, rebounding” (*n* = 81; 41% of ARFID); and Class 3 with “gradual response, low symptoms” (*n* = 57; 26% of ARFID). Class 3 had lower anxiety and R‐ED symptoms than Classes 1 and 2. Class 2 presented with older age than Class 1.

**Discussion:**

No ARFID‐specific classes emerged, underscoring similarities in response to intensive treatment between AN‐R and ARFID.

**Summary**

- Avoidant/restrictive food intake disorder (ARFID) and anorexia nervosa (AN) are both low‐weight restrictive eating disorders, but dietary restriction in ARFID is not motivated by body image.
- To understand how restriction differs between diagnoses, we examined trajectories of change in restriction during and after treatment.
- Three subgroups (“good response,” “good response, rebounding,” and “gradual response, low symptoms”) emerged that did not differentiate diagnoses, supporting transdiagnostic consideration of restriction across these diagnoses.

## 1 Introduction

Low‐weight restrictive eating disorders include avoidant/restrictive food intake disorder (ARFID) and anorexia nervosa (AN). AN treatment response is heterogeneous (Guarda 2008), with some individuals improving and others developing a chronic course, but much less is known about treatment response in ARFID. Prior research in AN has emphasized rapid versus non‐rapid responders to treatment (Linardon, Brennan, and De la Piedad Garcia 2016), but response may be more nuanced. Examining change trajectory using multiple timepoints—rather than relying on data at baseline and one end‐of‐treatment or naturalistic follow‐up timepoint—may aid treatment personalization.

Past work (Espel‐Huynh et al. 2020; Presseller et al. 2024, 2022) has examined latent trajectories of behavioral change during eating disorder (ED) treatment using latent growth mixture modeling (LGMM), a statistical method allowing for identification of subgroups with specific change trajectories. Espel‐Huynh et al. (2020) examined trajectories of change in overall ED symptoms in a mixed sample of AN, bulimia nervosa, binge eating disorder, and other specified feeding or eating disorder during residential ED treatment. They found gradual response, rapid response, and static response classes, supporting that binary distinctions between rapid and non‐rapid treatment responders may miss some trajectories of change. Presseller et al. (2022) examined trajectories of change in binge eating, compensatory behaviors, and regular eating among bulimia nervosa‐spectrum EDs during outpatient treatment and found static and moderate response trajectories of change in regular eating. While existing research supports heterogeneity in change in dietary restriction, prior studies focused on binge‐spectrum EDs or combined binge‐spectrum and restrictive EDs, which may differ on treatment response (Herpertz et al. 2011).

ARFID and AN are both characterized by dietary restriction, but are distinguished based on the absence of body image disturbance in ARFID. ARFID and AN are often treated in the same intensive care settings (Cooper et al. 2021; Ornstein et al. 2017), but the extent to which treatment response in ARFID is also heterogeneous and/or distinct from AN is unknown. No empirical classification studies that have sought to distinguish between ARFID and AN have examined how these diagnostic classes may be overlapping versus distinct over time (Richson et al. 2024). While some studies found similar outcome between AN and ARFID (Breithaupt et al. 2022; Bryson et al. 2018; Lange et al. 2019), others found worse outcome in ARFID (Forman et al. 2014; Vanzhula et al. 2023). While treatment for both ARFID and AN aims to decrease dietary restriction and increase body weight as necessary, it is possible that individuals with AN and ARFID may respond differently to interventions aimed at decreasing restriction (e.g., supervised meals/snacks, food exposures) due to differing underlying motivations for restriction. Characterizing diagnostic differences in trajectory of restriction change may advance understanding of restrictive ED nosology, as differing illness course can lend support to distinct diagnostic categories (Kendler 1990). Similar change trajectories in ARFID and AN may support transdiagnostic conceptualization of dietary restriction across these restrictive EDs, whereas distinct change trajectories may support continued categorical classification.

We examined latent trajectories of restriction change during treatment and follow‐up in low‐weight R‐EDs (ARFID and AN‐R). Specifically, we: (1) used LGMM to model change trajectories; (2) assessed clinical correlates of resulting class membership in order to understand factors that may be prospectively associated with trajectory of change and inform future research aiming to personalize treatment; and (3) tested differences in trajectory by diagnosis to understand whether ARFID and AN‐R differ on trajectory. We hypothesized > 1 class would emerge and that classes would differ on overall outcome and rate of restriction change. We expected that classes would differ on baseline characteristics, including demographic variables, duration of illness, ED symptoms, and comorbid symptoms, as these have been associated with treatment outcome (Haynos et al. 2021; Vall and Wade 2015). Finally, in line with current ED diagnostic criteria (American Psychiatric Association 2022) that consider AN and ARFID distinct disorders, we hypothesized that AN and ARFID would follow distinct courses, as evidenced by the emergence of ≥ 1 diagnosis‐specific class (i.e., a class with ≥ 90% of members falling into one diagnostic category).

## 2 Method

### 2.1 Participants

Adolescents and adults with low‐weight restrictive EDs (*n* = 276, 18% ARFID; 91% female; 84% White; 18% Hispanic/Latino/a; *M*_age_ = 18, SD_age_ = 6, age range = 11–56; *M*_BMI_ = 17.6 kg/m^2^, SD_BMI_ = 1.9 kg/m^2^) were recruited within a larger study examining naturalistic outcomes of a partial hospitalization ED program. All patients met standard admission criteria for ED treatment at a higher level of care (Yager et al. 2014). We only included individuals with the restricting subtype of AN given high overlap in core symptomatology between ARFID and AN‐R. In contrast, there is reduced alignment in behavioral symptoms between these disorders and binge‐purge AN. Specifically, there is evidence of increased ED symptoms (Reas and Rø 2018), greater comorbid symptoms (Casper et al. 1980; Garner, Garner, and Rosen 1993; Laessle et al. 1989), increased suicidality (Bulik et al. 2008; Forcano et al. 2011), greater impairment (Miranda‐Olivos et al. 2021; Mond et al. 2005), and poorer long‐term outcome (Steinhausen 2002; Støving et al. 2012; Ward et al. 2003) in AN‐BP relative to AN‐R, as well as some evidence for neurobiological differences between AN subtypes (Murao et al. 2017; Van Autreve et al. 2016). Further, in AN‐BP, clinicians may be targeting binge‐eating and/or purging behaviors in treatment in addition to restriction, which could influence trajectories of change in restriction. We did not include atypical AN based on the majority of ARFID participants in our sample (77%) presenting at an objectively low body weight at admission, consistent with the majority of our AN‐R participants presenting at an objectively low body weight (73%; the remainder had been at an objectively low body weight in the past 3 months and were considered to be AN‐R in partial remission). Inclusion in analyses also required participant data at ≥ 4 timepoints (see *Procedure* and *Statistical Analysis*). Participants (*n* = 160) with data at < 4 timepoints were older (*p* = 0.01), had a shorter length of stay (*p* < 0.001), and a higher admission BMI (*p* = 0.001), but did not differ on other variables of interest (*p*s > 0.05).

Of those with ARFID, 25% met criteria for the sensory sensitivity presentation, 52% for lack of interest, and 47% for fear of aversive consequences (presentations are not mutually exclusive). Presentation was missing for 18 ARFID participants, as this information was incorporated into assessment after data collection began.

### 2.2 Procedure

Program admission criteria aligned with American Psychiatric Association guidelines (Yager et al. 2014). See Brown et al. (2018) and Reilly et al. (2020), respectively, for adolescent and adult treatment programming and Richson et al. (2025) for ARFID‐specific programming. Following informed consent or parental consent plus assent, participants completed measures within 14 days of admission, 1 month into treatment, within 14 days prior to discharge, and 6 months and 1 year following discharge. Procedures were approved by the university institutional review board.

### 2.3 Measures

#### 2.3.1 Diagnostic Interviews

Semi‐structured interviews administered by trained bachelor's‐level research assistants and doctoral‐level trainees were used for ED diagnosis, current major depressive disorder diagnosis, and current anxiety disorder (i.e., panic disorder, specific phobia, agoraphobia, generalized anxiety disorder, or separation anxiety disorder) diagnosis. Assessors were supervised by licensed psychologists and attended diagnostic‐consensus meetings. Adults completed the Structured Clinical Interview for *DSM‐5* (First et al. 2015) or the MINI Neuropsychiatric Interview 7.0 (Sheehan et al. 1998); adolescents completed the Kiddie Schedule for Affective Disorders and Schizophrenia for School Age Children (Kaufman et al. 1997) or the MINI‐KID (Sheehan et al. 2010).

##### 2.3.1.1 ED Symptoms

The 45‐item Eating Pathology Symptoms Inventory (EPSI; Forbush et al. 2013) measured ED symptoms. This study used the Body Dissatisfaction, Cognitive Restraint, Restricting, and Excessive Exercise subscales, given theoretical relevance to low‐weight restrictive ED symptoms. Given differences in drivers of restriction between AN‐R and ARFID, we expected that any AN‐specific classes would have higher scores on Body Dissatisfaction, Cognitive Restraint, and Excessive Exercise than ARFID‐specific classes that emerged. The Restricting subscale had good internal consistency (α = 0.85–0.90) across timepoints. Other subscales had good internal consistency (α = 0.89–0.92).

##### 2.3.1.2 Depression Symptoms

The 9‐item Patient Health Questionnaire (PHQ‐9; Kroenke, Spitzer, and Williams 2001) measured depression symptoms. Internal consistency was good (α = 0.89).

##### 2.3.1.3 Anxiety Symptoms

The 20‐item trait scale of the State–Trait Anxiety Inventory (STAI; Spielberger et al. 1971) measured anxiety symptoms. Internal consistency was excellent (α = 0.92).

### 2.4 Statistical Analysis

LGMM was conducted in *R* using the ‘lcmm’ package (Proust‐Lima, Philipps, and Liquet 2015). EPSI‐Restricting scores were prenormalized prior to LGMM. One‐ to six‐class growth models were fitted with random intercepts and slopes of time to maximize model fit. Start values were based on the one‐class model. Each model was estimated 100 times. Linear and quadratic effects of time were estimated for each model. The best fitting solution was informed by the Bayesian information criterion (BIC; Schwarz 1978), sample size‐adjusted BIC (SABIC; (Sclove 1987)), Akaike information criterion (Bozdogan 1987), and entropy. When BIC, SABIC, and/or AIC disagreed, minimizing BIC was prioritized. Analyses required that participants had data at ≥ 4 timepoints (Frankfurt et al. 2016). In line with past work (Presseller et al. 2024, 2022), to further evaluate class separation, we also calculated the average posterior probability (i.e., probability that an observation belongs to the assigned class) and the percentage of cases with posterior probability ≥ 80% in the best‐fit solution.

Resulting classes were compared on clinical characteristics at admission, including demographics, ED treatment history, ED diagnosis, current anxiety and major depressive disorder prevalence, length of stay, duration of illness, ED symptoms (EPSI‐Body Dissatisfaction, EPSI‐Cognitive Restraint, and EPSI‐Excessive Exercise), and depression (PHQ‐9) and anxiety (STAI) symptoms using Chi‐square tests, Kruskal‐Wallis *H* tests, and ANOVAs. To further explore diagnostic differences, we compared AN‐R and ARFID on EPSI‐Restricting change scores. We also compared the proportion of ARFID presentations by class.

Data were examined for normality. Several variables (age, duration of illness, EPSI scores at admission and discharge) were not normally distributed; for these, Mann–Whitney *U* tests or Kruskal‐Wallis *H* tests were used for comparisons between diagnoses and between classes, respectively. For normally‐distributed variables, *t*‐tests and ANOVAs tested differences between diagnoses and classes, respectively. Welch‐corrected ANOVAs and contrasts are reported when indicated. Bonferroni correction was used to determine a family‐wise *p* value of 0.002 across our 23 validation variables of interest comparing classes (i.e., sex, race, ethnicity, percent who had received prior ED treatment, percent in each diagnostic category, percent of those with ARFID with each ARFID presentation, percent with a current anxiety disorder, percent with current major depressive disorder, age, length of stay, duration of illness, admission BMI, discharge BMI, EPSI‐Body Dissatisfaction, EPSI‐Cognitive Restraint, EPSI‐Restricting and change in EPSI‐Restricting from admission to each of the four other timepoints, EPSI‐Excessive Exercise, PHQ‐9, STAI) and of 0.006 across 9 validation variables of interest comparing diagnoses (EPSI‐Restricting score at each of the five timepoints, change in EPSI‐Restricting from admission to each subsequent timepoint). Listwise deletion was used to handle missingness on these comparisons given minimal missing data ranging from 0% to 1.4% on variables of interest.

Our primary analysis was intended to understand trajectories of change in restriction in behaviorally similar low‐weight disorders that differ primarily on the motivation for dietary restriction (i.e., AN‐R and ARFID). To understand whether our findings might also apply to a broader range of restrictive EDs that differ in weight status and/or binge eating and purging behaviors, we conducted sensitivity analyses including: (1) atypical AN and (2) atypical AN and AN‐BP in our models.

## 3 Results

### 3.1 Latent Growth Mixture Model

**FIGURE 1:**
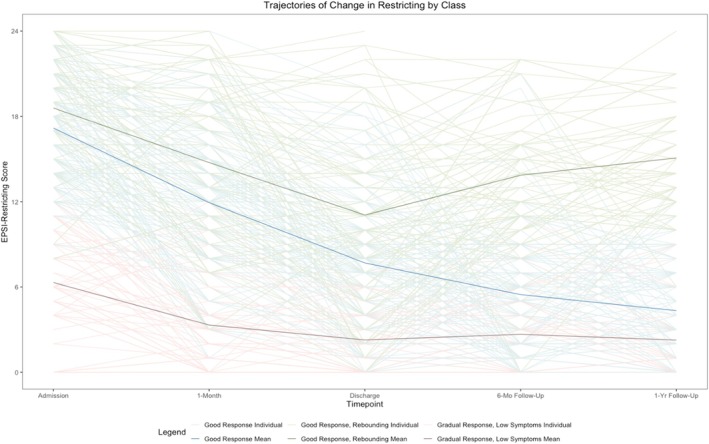
Trajectories of change in restriction during treatment and follow‐up by class. EPSI‐Restricting scores were standardized using the lcmm package in *R* prior to latent growth mixture modeling, but are represented here using unstandardized values to increase interpretability of scores (score range: 0–24).

A 3‐class solution emerged (Figure 1; see Table 1 for fit statistics): Class 1 comprising individuals with “good response” (*n* = 138); Class 2 with “good response, rebounding” (*n* = 81); and Class 3 with “gradual response, low symptoms” (*n* = 57). Individuals in the “good response” trajectory showed improvements in restriction during treatment that were maintained during follow‐up. Individuals in the “good response, rebounding” trajectory showed improvements in restriction during treatment, but increases in restriction during the follow‐up period. Individuals in the “gradual response, low symptoms” trajectory had lower mean restriction scores at admission that gradually improved during treatment and remained stable during the follow‐up period. Mean posterior probability for class membership was 88%. 77% of data points had posterior probability > 80%. All classes differed from one another on EPSI‐Restricting scores at all five timepoints (all *p*s < 0.001).

**TABLE 1:** Fit indices for LGMM.

	BIC	SABIC	AIC	Entropy
1 Class	4564.42	4532.71	4528.21	1.00
2 Classes	4548.06	4500.50	4493.76	0.75
**3 Classes**	**4520.89**	**4457.47**	**4448.48**	**0.74**
4 Classes	4537.41	4458.14	4446.90	0.66
5 Classes	4561.59	4466.46	4452.98	0.66
6 Classes	4583.03	4472.05	4456.31	0.68

*Note:* Best fit model bolded.

Abbreviations: AIC, Akaike information criterion; BIC, Bayesian information criterion; SABIC, Sample‐size adjusted BIC.

### 3.2 Correlates of Class Membership

Classes differed on diagnosis, duration of illness, ED symptoms, anxiety symptoms, and change in EPSI‐Restricting scores from admission to subsequent timepoints (Table 2). Class 2 (good response, rebounding) had a longer duration of illness than Classes 1 (good response) and 3 (gradual response, low symptoms). Classes 1 and 2 had higher levels of anxiety than Class 3. Class 1 had greater change in EPSI‐Restricting from admission to discharge, admission to 6‐month follow‐up, and admission to 1‐year follow‐up and higher levels of ED and anxiety symptoms than Class 3.

**TABLE 2:** Clinical characteristics by class.

	Class 1: Good response *n* = 138	Class 2: Good response, rebounding *n* = 81	Class 3: Gradual response, low symptoms *n* = 57	χ ^2^	*p*	Cramer's *V*
	*n* (%)	*n* (%)	*n* (%)			
Sex (% female)	129 (93%)	75 (93%)	47 (82%)	4.81	0.09	0.13
Race				6.00	0.42	0.11
White	116 (84%)	69 (85%)	46 (81%)			
Asian	6 (4%)	3 (4%)	3 (5%)			
Black	0 (0%)	2 (2%)	0 (0%)			
Other	14 (10%)	7 (9%)	8 (14%)			
Ethnicity (% Hispanic/Latinx)	21 (15%)	15 (19%)	14 (25%)	2.25	0.33	0.09
Previous ED treatment (% yes)	103 (75%)	59 (73%)	51 (89%)	5.30	0.07	0.14
ED Diagnosis (% ARFID)	17 (12%)	21 (26%)	13 (23%)	7.17	0.03	0.16
Current Anxiety Disorder	52 (38%)	44 (60%)	16 (11%)	10.52	0.01	0.20
Current Major Depressive Disorder	46 (33%)	35 (43%)	9 (16%)	14.01	0.03	0.17

	*M* (SD)	*M* (SD)	*M* (SD)	F or H	*p*	η ^2^
Age	18.1 (4.8)	20.1 (7.9)	16.8 (3.2)	12.01	0.01	0.02
Length of stay	104.1 (40.0)	113.3 (41.6)	101.42 (39.0)	1.81	0.17	0.01
Duration of illness	4.1 (5.2)^b^	6.5 (7.3)^a^, ^c^	3.7 (4.4)^b^	15.58	< 0.001	0.03
Admission BMI	17.5 (1.8)	17.5 (1.6)	18.0 (2.2)	1.47	0.23	0.01
Discharge BMI	20.4 (1.9)	20.2 (2.0)	20.7 (2.5)	1.15	0.32	0.01
EPSI body dissatisfaction	17.0 (7.3)^c^	17.7 (7.9)^c^	11.2 (7.8)^a^, ^b^	24.70	< 0.001	0.12
EPSI cognitive restraint	7.5 (4.3)	7.0 (4.8)	5.1 (4.0)	12.08	0.01	0.06
EPSI restricting						
Admission score	17.2 (3.6)^b^, ^c^	18.6 (4.0)^a^, ^c^	6.3 (3.6)^a^, ^b^	134.32	< 0.001	0.44
Admission to 1‐month change	−5.3 (5.1)	−3.9 (5.4)	−3.0 (4.3)	4.72	0.01	0.04
Admission to discharge change^d^	−9.5 (5.5)^c^	−7.6 (6.9)	−4.4 (4.4)^a^	21.53	< 0.001	0.10
Admission to 6‐month change	−11.7 (5.5)^b^, ^c^	−4.8 (6.3)^a^	−3.7 (4.5)^a^	55.82	< 0.001	0.30
Admission to 1‐year change	−12.6 (5.1)^b^, ^c^	−4.9 (6.3)^a^	−8.1 (6.7)^a^	72.49	< 0.001	0.40
EPSI excessive exercise	8.1 (7.0)^c^	7.9 (7.2)^c^	4.6 (5.6)^a^, ^b^	9.75	< 0.001	0.04
PHQ‐9	14.1 (6.2)	16.2 (6.1)	7.2 (6.0)	28.85	0.01	0.22
STAI	59.4 (10.8)^c^	61.5 (9.7)^c^	50.4 (11.2)^a^, ^b^	19.97	< 0.001	0.13

*Note: n* differs for current comorbidities, EPSI, PHQ‐9, and STAI as a result of missing data. After applying Bonferroni corrections, a significant *p* value was < 0.002.

Abbreviations: AN, anorexia nervosa; ARFID, avoidant/restrictive food intake disorder; BMI, body mass index (kg/m^2^); EPSI, Eating Pathology Symptoms Inventory; PHQ‐9, Patient Health Questionnaire 9‐item; STAI, State–Trait Anxiety Inventory.

^a^ Differs significantly from Class 1 (for posthoc comparisons).

^b^ Differs significantly from Class 2 (for posthoc comparisons).

^c^ Differs significantly from Class 3 (for posthoc comparisons).

^d^ Indicates unequal variances across groups.

### 3.3 Diagnostic Associations

Among ARFID, the proportion of those with the lack of interest presentation did not differ between classes after Bonferroni correction (χ ^2^ = 10.50, *p* = 0.03, Cramer's *V* = 0.40).

EPSI‐Restricting scores did not differ between AN‐R and ARFID at any timepoint (*p*s > 0.05, Figure S1). AN and ARFID did not differ in change in EPSI‐Restricting from admission to 1‐month (*t* = 2.20, *p* = 0.03, *d* = 0.35), discharge (*t* = 0.99, *p* = 0.32, *d* = 0.16), 6‐month follow‐up (*t* = 2.10, *p* = 0.04, *d* = 0.35), or 1‐year follow‐up (*t* = 3.01, *p* = 0.01, *d* = 0.54).

### 3.4 Sensitivity Analyses

We conducted two sensitivity analyses adding: (1) atypical AN cases and (2) atypical AN and AN‐BP cases into our model. In both analyses, BIC suggested a 1‐class solution was the best fit, while both SABIC and AIC suggested that a 6‐class solution was a better fit when including atypical AN (Table S1; Figure S2) and a 5‐class solution was a better fit when including atypical AN and AN‐BP (Table S2; Figure S3). Given our priority of minimizing BIC in ambiguous cases such as these, we selected the 1‐class solution as the best fit.

## 4 Discussion

We: (1) used LGMM to model trajectories of changes in dietary restriction in restrictive EDs; (2) assessed clinical correlates of latent class membership; and (3) tested differences in trajectory of change by diagnosis (AN‐R or ARFID). Three trajectories emerged: “good response,” “good response, rebounding,” and “gradual response, low symptoms.” The “good response, rebounding” class had longer duration of illness than Class 1 (“good response”) and Class 3 (“gradual response, low symptoms”), as well as higher baseline anxiety than Class 3. No diagnosis‐specific classes emerged, and after applying Bonferroni corrections, the proportion of participants with ARFID versus AN‐R did not differ by class.

Our findings are somewhat consistent with prior work using similar methodology. Espel‐Huynh et al. (2020) found gradual response, rapid response, and static response classes in a mixed sample of binge‐spectrum and EDs. Follow‐up data were not included, leaving unclear whether any classes had a rebounding trajectory; however, the static response class had lower initial symptoms similar to our “gradual response, low symptoms” class and the gradual response class had higher initial symptoms than the rapid response class. While rate of change in our “good response” and “good response, rebounding” classes did not differ significantly, lower levels of symptoms in the “good response” class at discharge align with Espel‐Huynh et al. (2020).

Longer duration of illness and higher anxiety differentiated those assigned to a class with a less favorable outcome (“good response, rebounding”) compared to a more favorable outcome (“good response” or “gradual response, low symptoms”). This is consistent with past work suggesting that duration of illness (Glasofer et al. 2020; Meule et al. 2023), age (Fichter et al. 2017; Meule et al. 2023), and comorbid symptoms (Derissen et al. 2023; Voderholzer et al. 2016) are associated with worse outcome in AN. Future research should test whether interventions that directly target anxiety could improve outcomes for individuals with these features.

The proportion of ARFID diagnoses did not differ by class. Though Class 1 comprised relatively few (12%) ARFID diagnoses, one‐third of those in the sample with an ARFID diagnosis (33%) were assigned to this class, suggesting that this trajectory is not specific to AN‐R. Thus, although drivers of restriction differ between ARFID and AN‐R, findings did not support diagnosis‐specific trajectories. Findings preliminarily support transdiagnostic study of dietary restriction and suggest that individuals with AN‐R and ARFID can be effectively treated within the same center when diagnostically‐specific programming is implemented.

When we broadened our sample to include atypical AN or both atypical AN and AN‐BP, models produced somewhat ambiguous solutions, with one‐class solutions appearing marginally more appropriate than five‐ or six‐class solutions, respectively. These solutions would suggest that changes in restriction during intensive treatment may be relatively consistent across AN‐R and ARFID diagnoses, in contrast to the findings from our primary analysis that suggests heterogeneity in treatment response. Thus, our findings suggesting heterogeneity in treatment response may be constrained to low‐weight restrictive EDs that are not characterized by binge‐eating and purging behaviors. Of note, the average trajectory in both of these supplemental models is similar to the “good response” class in our primary analysis. The “good response” class was the largest class in the primary analyses. It is possible that a large proportion of individuals with AN‐BP and atypical AN similarly improved with treatment and maintained these reductions in restriction during follow‐up. Thus, the addition of a large number of individuals with a similar trajectory to the “good response” class may have masked the presence of other classes in these models and thus resulted in a more homogeneous one‐class solution. Further testing of these trajectories in other samples and across restrictive ED diagnostic groups is necessary.

Study strengths include a large clinical sample, multiple follow‐up timepoints, and use of LGMM to empirically determine trajectories of dietary restriction change in two phenotypically similar disorders, ARFID and AN‐R. However, though our data permitted LGMM (Frankfurt et al. 2016), the number of data points available per participant was modest. Future research might prioritize gathering more granular data through ecological momentary assessment or weekly outcome tracking. Further, we acknowledge that attrition from study follow‐up assessments was high. While the EPSI‐Restricting subscale assesses restriction independent of the motivation for said restriction, we acknowledge the EPSI was not developed to capture ARFID pathology. Ethnoracial and gender diversity is limited in our sample, which may be particularly relevant to generalizability given higher rates of ARFID (relative to other EDs) in males (Katzman et al. 2021). When considering our sensitivity analyses, our findings may not generalize to AN‐BP and atypical AN. Finally, the present study examined only changes in dietary restriction during treatment; future research may seek to understand trajectories of changes in other constructs relevant to restrictive EDs, such as global ED symptoms, comorbid mood and anxiety symptoms, emotion regulation, and impairment.

We found three trajectories of change in dietary restriction in AN‐R and ARFID throughout treatment and follow‐up: “good response,” “good response, rebounding,” and “gradual response, low symptoms.” These trajectories mapped onto unique clinical features. Findings may reveal risk factors for relapse following intensive treatment and could help inform targets of post‐discharge care. Overall, findings did not support wholly distinct trajectories of change in ARFID relative to AN‐R.

## Author Contributions

**Sophie R. Abber:** conceptualization, formal analysis, methodology, writing – original draft, writing – review and editing. **Emily K. Presseller:** conceptualization, methodology, writing – review and editing. **Brianne N. Richson:** conceptualization, writing – review and editing. **Thomas E. Joiner:** writing – review and editing. **Christina E. Wierenga:** funding acquisition, methodology, resources, supervision, writing – review and editing.

## Conflicts of Interest

The authors declare no conflicts of interest.

## IRB Statement

The authors assert that all procedures contributing to this work comply with the ethical standards of the relevant national and institutional committees on human experimentation and with the Helsinki Declaration of 1975, as revised in 2008. The current study was approved by the University of California, San Diego's Institutional Review Board (180055).

## Supporting information


**Data S1.** Supporting Information.

## Data Availability

The data that support the findings of this study are available from the corresponding author upon reasonable request.
