# Supplementary material for: Latent Trajectories of Change in Dietary Restriction During Treatment in Avoidant/Restrictive Food Intake Disorder and Anorexia Nervosa
Source: Int J Eat Disord. 2025 Jan 20;58(4):748–55. doi: 10.1002/eat.24382 (PMC11969032; doi:10.1002/eat.24382)
Supplement: Supplementary file 1 — Data S1. Supporting Information. [file EAT-58-748-s001.docx]

**Figure S1.** Trajectories of change in restriction during treatment and follow-up by diagnosis in ARFID, AN-R, AN-BP, and atypical AN.


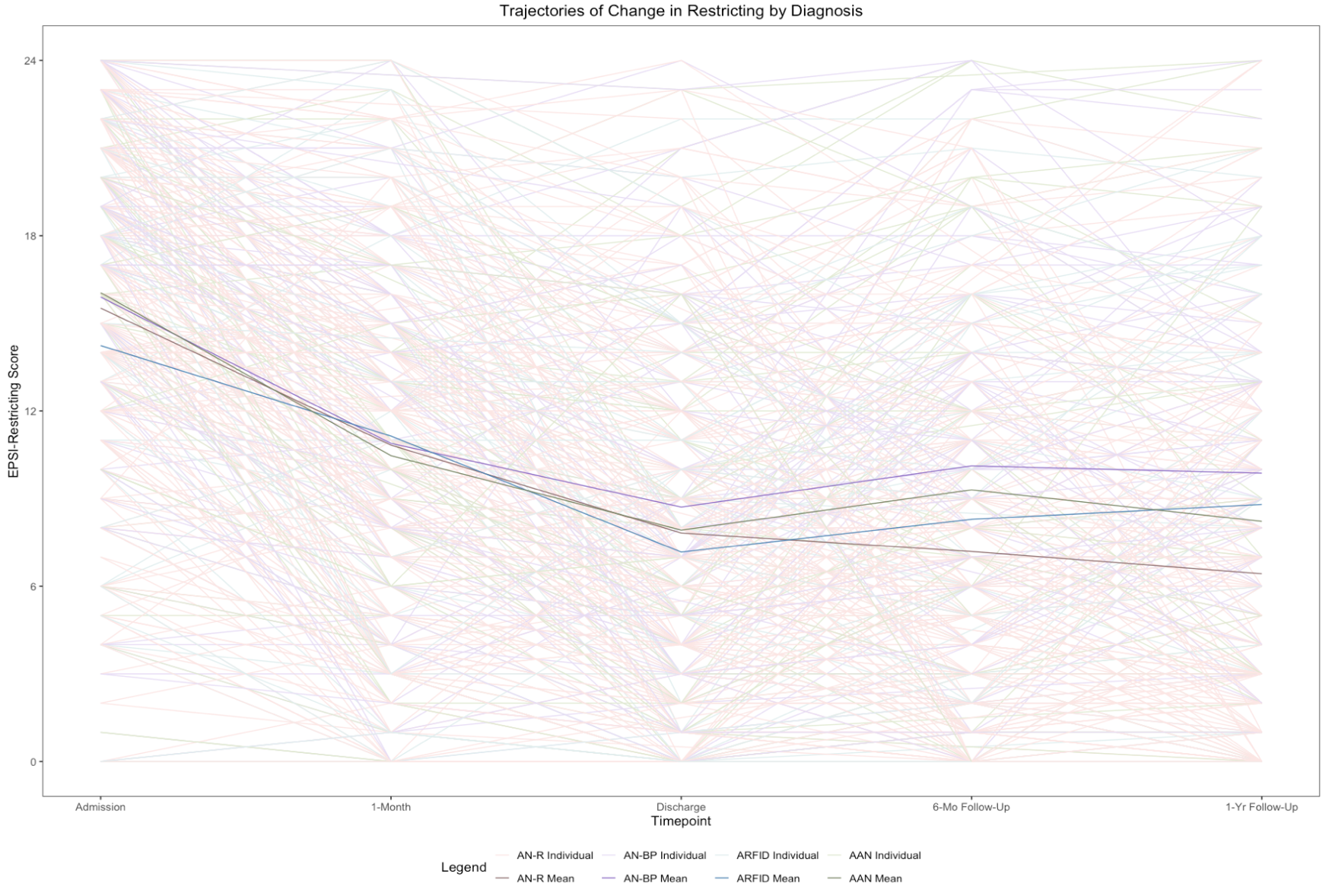


*Note.* EPSI-Restricting scores were standardized using the *lcmm* package in R prior to latent growth mixture modeling, but are represented here using unstandardized values to increase interpretability of scores (score range: 0-24).

**Table S1.** Fit indices for LGMM including ARFID, AN-R, and atypical AN.

|  | BIC | SABIC | AIC | Entropy |
| --- | --- | --- | --- | --- |
| 1 Class | **6444.57** | 6412.84 | 6406.25 | 1.00 |
| 2 Classes | 6452.11 | 6404.53 | 6394.63 | .63 |
| 3 Classes | 6460.49 | 6397.04 | 6383.85 | .72 |
| 4 Classes | 6458.68 | 6379.37 | 6362.88 | .66 |
| 5 Classes | 6474.08 | **6378.91** | **6359.12** | .70 |
| 6 Classes | 6501.45 | 6390.42 | 6367.33 | .63 |

*Note.* BIC=Bayesian Information Criterion, SABIC=Sample-Size Adjusted BIC, AIC=Akaike Information Criterion. Lowest values of each fit index are bolded.

**Figure S2.** Trajectory of change in restriction during treatment and follow-up in the one-class solution for the model including ARFID, AN-R, and atypical AN.


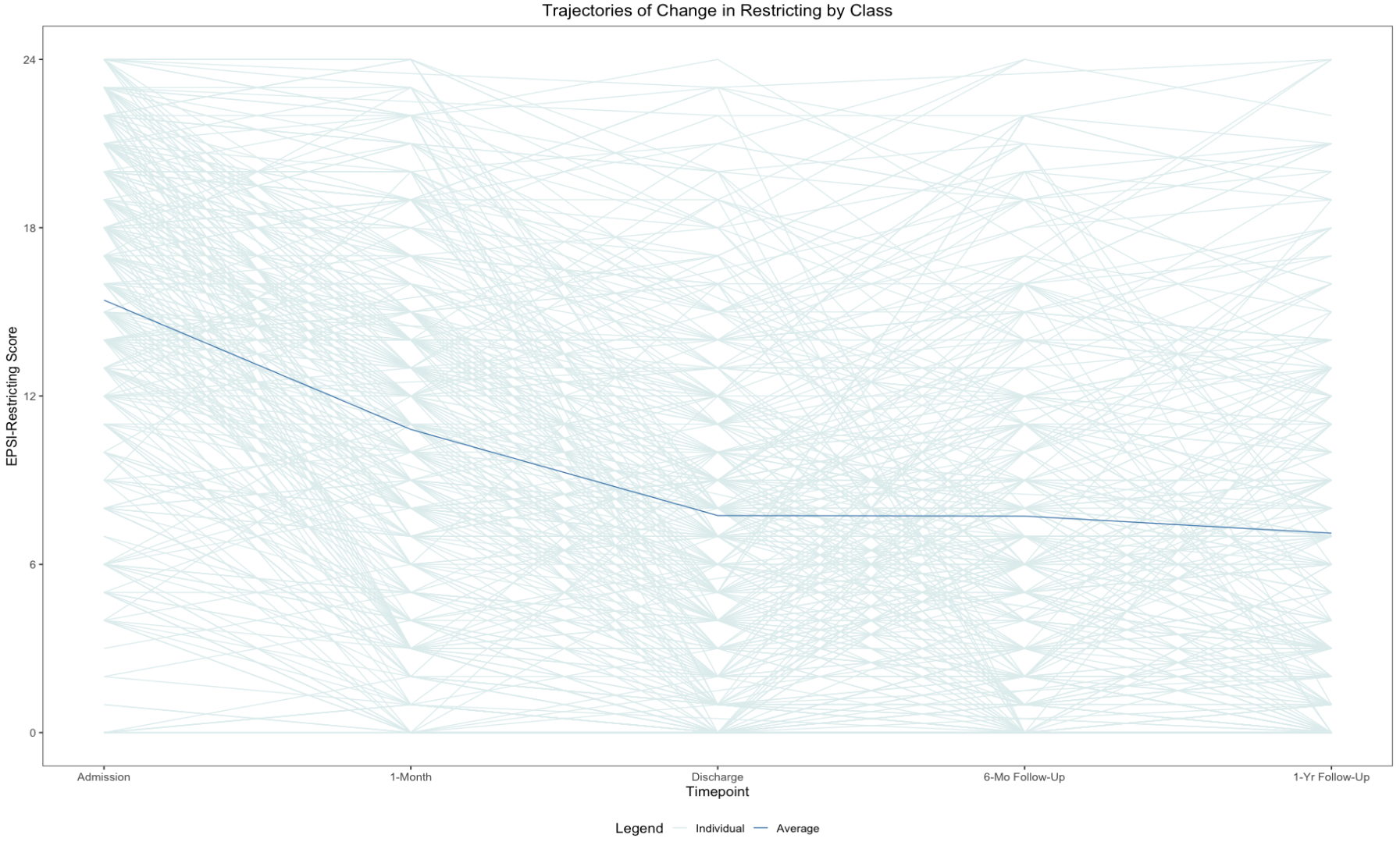


*Note.* EPSI-Restricting scores were standardized using the *lcmm* package in R prior to latent growth mixture modeling, but are represented here using unstandardized values to increase interpretability of scores (score range: 0-24).

**Table S2.** Fit indices for LGMM including ARFID, AN-R, atypical AN, and AN-BP.

|  | BIC | SABIC | AIC | Entropy |
| --- | --- | --- | --- | --- |
| 1 Class | **7867.63** | 7835.90 | 7827.09 | 1.00 |
| 2 Classes | 7877.77 | 7830.17 | 7816.95 | .49 |
| 3 Classes | 7883.55 | 7820.08 | 7802.46 | .58 |
| 4 Classes | 7897.83 | 7818.49 | 7796.47 | .60 |
| 5 Classes | 7922.64 | 7827.44 | 7801.01 | .64 |
| 6 Classes | 7926.18 | **7815.12** | **7784.28** | .59 |

*Note.* BIC=Bayesian Information Criterion, SABIC=Sample-Size Adjusted BIC, AIC=Akaike Information Criterion. Lowest values of BIC, SABIC, and AIC are bolded.

**Figure S3.** Trajectory of change in restriction during treatment and follow-up in the one-class solution for the model including ARFID, AN-R, AN-BP, and atypical AN.


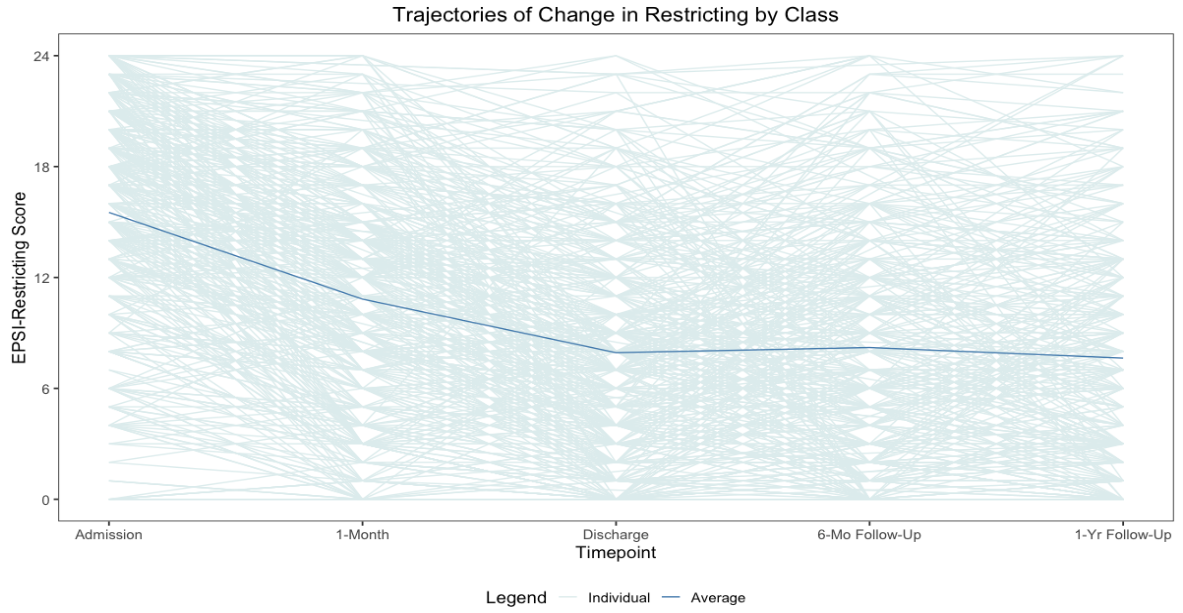


*Note.* EPSI-Restricting scores were standardized using the *lcmm* package in R prior to latent growth mixture modeling, but are represented here using unstandardized values to increase interpretability of scores (score range: 0-24).
